# A cross sectional study between the prevalence of chronic pain and academic pressure in adolescents in China (Shanghai)

**DOI:** 10.1186/s12891-015-0625-z

**Published:** 2015-08-22

**Authors:** Yongxing Zhang, Guoying Deng, Zhiqing Zhang, Qian Zhou, Xiang Gao, Liqing Di, Qianzi Che, Xiaoyu Du, Yun Cai, Xuedong Han, Qinghua Zhao

**Affiliations:** Department of Orthopaedics, Shanghai First People’s Hospital, School of Medicine, Shanghai Jiao tong University, Shanghai, 200080 China; Department of General Surgery, Huai’an First People’s Hospital Affiliated to Nanjing Medical University, Huai’an, Jiangsu Province 223300 China

## Abstract

**Background:**

The purpose of this study was to investigate the prevalence of four types of chronic pain (headache, abdominal pain, neck and shoulder pain (NSP), and low back pain (LBP)) and to explore the relationship between the prevalence of chronic pain and self-reported academic pressure in high school students in Shanghai, China.

**Method:**

Three thousand students were randomly surveyed on related issues using a questionnaire, and the results were analyzed using a multivariate logistic regression model.

**Results:**

Among the 2849 high school students who completed the questionnaire, the overall prevalence rates of headache, abdominal pain, NSP, and LBP were 30.3, 20.9, 32.8, and 41.1 %, respectively. The students in general experienced a heavy burden of learning, a high level of stress, and sleep deprivation, which were closely related to the four types of chronic pain.

**Conclusion:**

Chronic pain is a common condition in Chinese adolescents and is closely related to self-reported academic pressure.

## Background

Chronic pain can be defined as an individual’s subjective perception of an unpleasant feeling lasting at least 3 months [1]. Chronic pain directly affects quality of life and work efficiency and imposes huge economic burden on families and society [2, 3]. In the United Kingdom, back pain accounts for £1632 million in direct expenditure and £10,668 million in indirect costs every year. In the United States, over 80 million people suffer from chronic pain, and more than $90 billion is spent on medical expenses, legal proceedings, and unemployment and disability benefits every year [4]. It has been preliminarily estimated that more than 300 million people in China suffer from chronic pain, and the number increases at a rate of 10–20 million people per year. The prevalence rate of chronic pain ranks second only to the common cold and is much higher than that of malignant tumors, hypertension, and diabetes [5]. Many types of chronic pain originate from puberty [6]. For example, the prevalence of frequent headache and migraine was reported 24.83 % in the adolescents of Campinas, Brazil [7], and a survey in Finland showed that NSP and LBP occurred at least once a week in respectively 26 and 12 % of 14- to 18-year-olds [8]. In spite of different criterion, however, the statistics can still be significant to show the current situation of adolescent health.

In addition to chronic pain, academic pressure also besets Chinese adolescents. Data published by the Ministry of Education of the People’s Republic of China have shown that nearly 10 million students participated in the annual national college entrance exam each year between 2008 and 2013. Maybe unlike the adolescents of Western developed countries, Chinese adolescents face both academic and mental pressures due to intense competition, which results in unique living habits and psychological states. Depression is closely associated with certain types of chronic pain [6, 7]. The prevalence of chronic pain is rather high in the adolescent population [8], which is closely related to poor mental state and can be easily affected by various factors, including psychological factors and living habits [9]. Depression and poor mental state are also high-risk factors for low back pain (LBP) in adults of working age [10, 11]. Although previous studies have confirmed that education-related stress increases the risk of developing LBP in adolescents [12], the correlation between the academic pressure that Chinese adolescents experience currently and the prevalence with chronic pain has yet to be explored. This study investigated the prevalence of chronic pain and the current academic pressure situation in adolescents in China (Shanghai) and examined the relationship between these factors. By investigating the current academic pressure situation in Chinese adolescents and analyzing the relationships between various learning-burden-related risk factors and different types of chronic pain, we hope that this study may provide a reference for improving adolescent health in the future.

## Methods

All of the schools, parents, and students involved in the study provided their written informed consent before participating in the survey. The study protocol was reviewed and approved by the Ethics Committee of the School of Medicine, Shanghai Jiaotong University.

### Subjects of study

A total of 30 high schools were randomly selected from the 237 full-time ordinary high schools registered in Shanghai, and 100 students with age various from 16 to 18 were chosen from each school using a random selection method based on the students’ identification numbers to complete the survey questionnaire. Due to the unique academic circumstances of the twelfth grade year, only a small portion of the students completed the questionnaire.

### Research methods

Because the subjects of the survey were high school adolescents (including high school seniors) whose mental states were less restrained and less controllable, follow-up was rather difficult. Cohort study was not suitable for the analysis of the survey results. Therefore, cross-sectional study was conducted instead.

The questionnaire was designed with reference to the related literature. Based on the problems experienced by adolescents as discovered through interviews, a question bank was established. The questions were modified to incorporate the characteristics of high school students.

To ensure the validity of the questionnaire, a pre-survey was conducted among 120 college freshmen before the launch of the formal survey. Based on the results of the pre-survey, the survey questionnaire was modified to eliminate duplication and to remove the factors that had little correlation with disease prevalence (measurement of sampling adequacy MSA <0.50). In addition, the logic of each question was ensured so that the survey participants would understand the questions and take choices correctly. The final Kaiser-Mayer-Olkin (KMO) index of the questionnaire was 0.593. The questionnaire typically took less than 20 min to complete.

Thirty undergraduates from the Department of Clinical Medicine at the School of Medicine, Shanghai Jiao Tong University were responsible for distributing and retrieving the questionnaires. The undergraduates had received training related to aspects of the survey in advance. Before conducting the survey, the undergraduates gave the participants a popular science lecture; marked the specific scope of headache, abdominal pain, neck and shoulder pain (NSP), and LBP using diagrams of the human body; and explained in detail the characteristics of pain and the differences between post-exercise soreness, menstrual pain in women, and post-traumatic pain. Onset of pain was defined as pain lasting for more than 10 min, and we define “chronic pain” as “the pain lasting over 6 h single a time or short time with high frequency over 2–3 one day, and this bad situation happened more than 3 times in one month”. But in the questionnaire, simply we described the standard as” in the last 3 months, how often did you feel this kind of frequent or continuous pain in neck/shoulder, low back, head and abdomen” to differentiate chronic pain from acute pain. Instead of directly using “yes” and “no” to assess the exposure to risk factors, the onset frequency of pain was classified into the following four levels: “almost never”, less than once per month; “occasionally”, 1–3 times per month; “often”, 1–3 times per week; and “always”, more than 3 times per week. General treatment of the results: “often” and “always” were treated as “yes”, while the other two levels, “almost never” and “occasionally”, were treated as “No”.

Two weeks after the completion of the large-scale questionnaire survey, 50 participants were randomly selected for a two-factor test-retest reliability study. The test-retest reliability ranged between 0.69 and 0.81, which is measured by Kappa.

### Data statistics and analysis

Except for the gender and grade year of the survey participants, the contents of the survey were divided into two major parts, direct and indirect indicators, which are all included in Table 1, 4 (A and B) and 5.

**Table 1:** Demographic factors and chronic pain in high school students

	N	% With NSP	P value	OR (95 % CI)	% With LBP	P value	OR (95 % CI)	% With headache	P value	OR (95 % CI)	% With AP	P value	OR (95 % CI)
Gender			<0.001			0.001			<0.001			0.029	
Male	1254	36.8 %		0.69 (0.58–0.82)	29.0 %		0.74 (0.61–0.88)	24.6 %		0.54 (0.45–0.65)	18.5 %		0.79 (0.64–0.98)
Female	1333	44.1 %		1	35.4 %		1	35.0 %		1	21.5 %		1

*NSP* neck/shoulder pain, *LBP* low back pain, *AP* abdominal pain, *OR* odds ratio, *CI* confidence interval

#### Direct indicators

The indicators that reflected the amount of learning burden included “extracurricular learning tasks,” “average daily sitting time” and “academic ranking”. Self-reported feelings of the adolescents, such as “very tired after everyday learning” and “not enough rest,” were used as indicators to reflect the direct impact of the burden of learning.

#### Indirect indicators

We regard living habits such as the length and quality of sleep, playing with musical instruments, smoking/drinking or not, as the indirect indicators of learning burden. Additionally, China ranks second in the world in the percentage of glasses-wearing adolescents and lags behind more than one hundred countries in average sleep time per day. These phenomena are likely related to the tremendous academic pressure Chinese adolescents have faced [13]. Changes in living habits caused by learning burden also reflect the academic pressure that adolescents experience. Therefore, we inquired about vision conditions in adolescents with questions such as “whether the adolescent became nearsighted”, “how long the adolescent had suffered from nearsightedness”, and “when nearsightedness started”. In addition, questions regarding “recess,” “self-feeling and exercise time”, and “sleep time” were used to determine the adolescents’ time allocation.

Data analysis was performed using the SPSS 21.0 software (SPSS Inc., Chicago, IL). Uncompleted questionnaires, questionnaires containing obvious errors (i.e., the errors were not caused by the answer choices of the questions), and the questionnaires filled out with answers containing apparent logic errors were excluded. The candidate risk factors were examined using a multivariate logistic regression model that included all the candidate risk factors. Backward stepwise regression procedure was used and the threshold for variant removal was set at 0.10. The results were presented using the odds ratio (OR) values and 95 % confidence intervals (CI). A P value less than 0.05 was considered statistically significant.

## Results

In this study, a total of 3000 questionnaires were handed out, of which 2849 questionnaires (95 %) were successfully retrieved and 2587 questionnaires (86.2 %) were valid (if there are more than 15 % of the answers which cannot reflect the participant’s purpose about this questionnaire, then we regard this kind of questionnaires we have collected as invalid ones). Among the 262 unqualified questionnaires, 110 of them were excluded because they were far from complete and 5 questionnaires were abandoned due to lack of response to key questions. Analysis of the remaining 147 uncompleted questionnaires showed that the results regarding the prevalence rates of the four types of chronic pain were not statistically significantly different from the results of the 2587 completed questionnaires.

The overall prevalence rates of headache, abdominal pain, NSP, and LBP were 30.3, 20.9, 41.1, and 32.8 %, respectively, among the ordinary high school students in Shanghai. The prevalence rates of all four types of chronic pain were elevated in female students compared to male students (headache, 35.00 % *vs.* 24.60 %; abdominal pain, 21.50 % *vs.* 18.50 %; NSP, 44.10 % *vs.* 36.80 %; and LBP, 35.40 % *vs.* 29.00 %), and the differences were statistically significant (Table 1).

### Academic pressure currently faced by Chinese adolescents

#### Direct indicators

The survey results showed that 34.7 % of the high school students spent an average of more than 10 h per day studying/sitting; 58.9 % of the students continued studying after school for more than 2 h; 44.2 % of the students had regular extracurricular learning tasks (such as various training courses and remedial classes); and 32.0 % of the students felt that they failed to achieve their daily learning goals (Table 2). The self-reported academic pressure included not only the heavy burden of learning but also tremendous mental stress: 60.7 % of the high school students frequently felt tired after everyday learning, and 32.2 % of students received much pressure from parents and teachers.

**Table 2:** Direct indicators of burden of learning

	Frequency	Percentage
Feel much pressure from parents and teachers		
No	1755	67.8 %
Yes	832	32.2 %
Have a good relationship with other students		
No	479	18.5 %
Yes	2108	81.5 %
Finish everyday learning tasks		
No	827	32.0 %
Yes	1760	68.0 %
Feel satisfied with myself		
No	1247	48.2 %
Yes	1340	51.8
I have extracurricular learning tasks		
No	1443	55.8
Yes	1144	44.2
I feel very tired after everyday learning		
Never	173	6.7
Occasionally	844	32.6
Often	983	38.0
Always	587	22.7
Your rank at school		
<25 %	703	27.2 %
25–50 %	896	34.6 %
50–75 %	683	26.4 %
>75 %	305	11.8 %
How long do you need to continue studying after school?		
<1 h	637	24.6
1–2 h	424	16.4
2–3 h	855	33.0
>3 h	671	25.9
How long do you study every day?		
<4 h	341	13.2
4–6 h	288	11.1
6–8 h	445	17.2
8–10 h	616	23.8
>10 h	897	34.7

#### Indirect indicators

The study showed that 68.1 % of the ordinary high school students in Shanghai wore glasses for daily activities (Table 3). In addition, the survey showed that academic pressure led to changes in living habits: 52.0 % of the students fell asleep after 11:00 pm; 65.0 % of the students were sleep-deprived; and 54.5 % of the students had poor-quality sleep and frequently suffered from insomnia and excessive dreaming.

**Table 3:** Indirect indicators of burden of learning

	Frequency	Percentage
I wear glasses		
No	824	31.9
Yes	1763	68.1
How long have you had bad eyesight?		
Since senior high school	348	13.5
Since junior high school	1322	51.1
Since before the above	526	20.3
My eyesight has always been good	391	15.1
How far is the book from your eyes?		
<15 cm	505	19.5
15–20 cm	1112	43.0
20–25 cm	729	28.2
>25 cm	241	9.3
I can smell smoke (secondhand smoke or smoking)		
No	1703	65.8
Yes	884	34.2
I drink alcohol		
No	2289	88.5
Yes	298	11.5
Do you often play a musical instrument?		
No	2036	78.7
Yes	551	21.3
Usual sleeping time		
Before 11:00 p.m.	1243	48.0
11:00–12:00 p.m.	1037	40.1
12:00 p.m.–1:00 a.m.	236	9.1
After 1:00 a.m.	71	2.7
I can fall asleep but don’t get enough sleep		
No	905	35.0
Yes	1682	65.0
I’m an insomniac and have too many dreams		
No	1177	45.5 %
Yes	1410	54.5 %

### Current prevalence of chronic pain in Chinese adolescents

#### Direct indicators (Table 4A and B)

**Table 4:** Direct indicators of burden of learning and chronic pain in high school students

N		% With NSP	P value	OR (95 % CI)	% With LBP	P value	OR (95 % CI)	% With headache	P value	OR (95 % CI)	% With AP	P value	OR (95 % CI)
(A)													
Q1: Do you feel much pressure from parents and teachers?													
			<0.001			<0.001			<0.001			<0.001	
No	1755	34.7 %		1	26.9 %		1	23.6 %		1	16.6 %		1
Yes	832	52.9 %		1.93 (1.61–2.32)	43.8 %		1.89 (1.57–2.28)	43.3 %		2.26 (1.86–2.74)	27.4 %		1.73 (1.41–2.14)
Q2: Do you have a good relationship with other students?													
				N		0.01			<0.001			0.004	
No	479	39.0 %			35.7 %		1	36.7 %		1	24.4 %		1
Yes	2108	40.9 %			31.5 %		0.74 (0.60–0.93)	28.4 %		0.62 (0.50–0.78)	19.1 %		0.70 (0.55–0.89)
Q3: Do you have extra learning tasks after class?													
			0.001			0.002			<0.001				
No	1443	36.5 %		1	28.8 %		1	26.2 %		1	19.4 %		N
Yes	1144	45.6 %		1.33 (1.12–1.58)	36.8 %		1.33 (1.11–1.59)	34.7 %		1.44 (1.20–1.73)	20.9 %		
(B)													
Q4: Do you feel very tired after school?													
			<0.001			<0.001			<0.001			<0.001	
No	1017	25.2 %		1	18.7 %		1	16.9 %		1	13.0 %		1
Yes	1570	50.5 %		2.72 (2.28–3.26)	41.1 %		2.76 (2.27–3.36)	38.4 %		2.63 (2.15–3.21)	24.6 %		1.99 (1.59–2.49)
Q5: How long have you been studying every day?													
			<0.001			<0.001			0.006			0.086	
<4 h	341	43.7 %		1	33.4 %		1	29.3 %		1	18.2 %		1
4–6 h	288	45.5 %		0.92 (0.65–1.28)	41.3 %		1.26 (0.89–1.77)	30.2 %		0.95 (0.66–1.37)	22.2 %		1.18 (0.79–1.77)
6–8 h	445	32.1 %		0.60 (0.44–0.82	19.6 %		0.48 (0.34–0.68)	20.0 %		0.67 (0.47–0.94)	15.7 %		0.86 (0.59–1.27)
8–10 h	616	36.0 %		0.64 (0.48–0.86)	28.6 %		0.73 (0.54–0.98)	30.4 %		0.95 (0.70–1.29)	18.3 %		0.95 (0.67–1.36)
>10 h	897	45.0 %		0.96 (0.74–1.26)	37.9 %		1.15 (0.87–1.53)	34.8 %		1.16 (0.86–1.55)	23.4 %		1.26 (0.91–1.75)
Q6: What is your rank in school?													
			<0.001			0.003			0.006			0.026	
<25 %	703	36.1 %		1	27.7 %		1	28.2 %		1	16.6 %		1
25 %–50 %	896	42.1 %		1.45 (1.17–1.81)	33.3 %		1.46 (1.16–1.84)	27.7 %		1.08 (0.85–1.36)	20.3 %		1.33 (1.02–1.73)
50 %–75 %	683	39.2 %		1.14 (0.90–1.44)	32.5 %		1.26 (0.98–1.61)	30.2 %		1.08 (0.84–1.40)	20.5 %		1.22 (0.92–1.62)
>75 %	305	49.2 %		1.71 (1.28–2.30)	39.7 %		1.62 (1.19–2.20)	40.3 %		1.70 (1.25–2.32)	26.2 %		1.66 (1.18–2.33)

*NSP* neck/shoulder pain, *LBP* low back pain, *AP* abdominal pain, *OR* odds ratio, *CI* confidence interval

Among the high school students with extracurricular learning tasks, 45.60 % suffered from NSP, 36.80 % suffered from LBP, 34.70 % suffered from headache, and 20.9 % suffered from abdominal pain. Among the high school students who studied more than 10 h every day, the percentages of students with NSP, LBP, headache, and abdominal pain were 45.00, 37.90, 34.80, and 23.40 %, respectively. In students whose academic grades ranked in the lower quartile, the prevalence rates of NSP, LBP, headache, and abdominal pain were 49.20, 39.70, 40.30, and 26.20 %, respectively. The prolonged study time, heavy learning burden, and much pressure from parents, teachers and classmates were closely associated with the prevalence of chronic pain.

#### Indirect indicators (Table 5)

**Table 5:** Indirect indicators of burden of learning and chronic pain in high school students

N		% With NSP	P value	OR (95 % CI)	% With LBP	P value	OR (95 % CI)	% With headache	P value	OR (95 % CI)	% With AP	P value	OR (95 % CI)
Q1: Do you often play a musical instrument, such as piano or violin?													
			0.004			0.003			N			0.001	
No	2036	39.2 %		1	31.1 %		1	29.4 %			19.1 %		1
Yes	551	45.4 %		1.35 (1.10–1.66)	36.8 %		1.39 (1.12–1.72)	31.9 %			23.8 %		1.48 (1.17–1.87)
Q2: When do you usually get to bed at night?													
			N			N			0.013			0.023	
Before 23:00	1243	39.2 %			31.6 %			27.3 %		1	18.5 %		1
23:00–24:00	1037	42.0 %			31.1 %			30.3 %		1.09 (0.90–1.33)	19.5 %		0.99 (0.80–1.23)
24:00–1:00	236	41.5 %			38.1 %			38.6 %		1.29 (0.94–1.77)	27.5 %		1.35 (0.96–1.88)
After 1:00	71	39.4 %			42.3 %			43.7 %		2.26 (1.33–3.84)	31.0 %		2.04 (1.17–3.53)

*NSP* neck/shoulder pain, *LBP* low back pain, *AP* abdominal pain, *OR* odds ratio, *CI* confidence interval

For students who went to sleep after 1:00 a.m., the prevalence rates of headache and abdominal pain were 43.70 and 31.00 %, respectively, which were significantly higher than in the students who slept earlier. However, no correlation was found between wearing glasses and the prevalence of chronic pain in this study.

## Discussion

This study is the first in China to investigate the correlation between the self-reported academic pressure that Chinese adolescents face currently and the prevalence of chronic pain (headache, abdominal pain, NSP, and LBP) using cross-sectional analysis. The survey results showed that the prevalence rates of headache, abdominal pain, NSP, and LBP in ordinary high school students in Shanghai China were 30.3, 20.9, 32.8, and 41.1 %, respectively, which might be higher than the results of international research surveys in spite of different definitions about pain and chronic pain [8, 14]. The high prevalence of chronic pain may be related to the current state of high school education in China, with characteristics such as prolonged study time, a heavy burden of learning, and huge pressure. Chinese students experience similar levels of academic pressure regardless of gender. However, the prevalence of chronic pain was elevated in female students compared to male students, which was consistent with the results of previous cross-sectional studies [14, 15]. The reasons for this phenomenon might include the following: (1) Compared to males, female adolescents are more emotionally sensitive [16] and feel fatigued more easily [17]. In addition, although we claim the difference between chronic pain and menstrual pain, it’s still difficult for them to distinct the two types of pains. Besides, primary dysmenorrhea is rather common among female adolescents. Therefore, female adolescents are more readily affected by academic pressure and complaining about pains. (2) Females have lower pain thresholds and tolerance levels than males of the same age [18–20]. Therefore, the symptoms of chronic pain are more likely to manifest in females [17]. The study also shows a correlation between many working hours and chronic pain, but only if the working hours is so long enough (e.g. >10 h) that the correlation shows significant. So is it with the correlation between rank in school and chronic pain——only if the gap of academic ranking is large enough (e.g. ranking at <25 % vs. >75 % of the students) that significance exists (Table 4B). The characteristics of chronic pain displayed as the grade year increased were consistent with the changes in the burden of learning. However, multivariate analysis indicated no significant correlation between grade and chronic pain, which is most likely due to the heavy learning burden generally experienced by the students in all grade years. But due to only a small portion of the students completed the questionnaire because of the unique academic circumstances of the twelfth grade year, this is just a speculation rather than a conclusion. It has been shown that there is a correlation between the mental states of Chinese adolescents and the prevalence of chronic pain [21]. Mental factors may result in varying degrees of pain sensitivity in adolescents [7]. Under Chinese education system, the test score is the most important criterion to evaluate a middle school student. Achieving acceptance into the nation’s top universities is the absolute mainstream ideology of the general public. Because of this ideology, Chinese adolescents may be burdened with much more mental pressure. However, self-reported academic pressure exerts distinct effects on different types of chronic pain in adolescents.

### Headache

Chronic headache is not a rare condition in adolescents [22]. A study in German showed that over 80 % of adolescents aged 13–18 years had self-reported headache symptoms in the last 6 months [22] (defined according to the German version of the International Classification of Headache Disorders (2nd Edition)). Worldwide, 54.4 % of the adolescent population suffers from headaches [23]. This study showed that 30.3 % of Chinese adolescents experienced headaches more than once per week in the last 3 months. Moreover, the prevalence of chronic headache was closely related to the average daily study time, extracurricular learning tasks, and stress received from parents, classmates, and teachers. The reason may be that heavy academic pressure induces an increase in risk factors for headaches (elevated levels of mental stress, lack of sleep, and less exercise) [24] and changes in adolescents’ living habits (smoking, alcohol and coffee consumption), perhaps promoting the prevalence of chronic headache nonspecifically [25].

### Abdominal pain

Chronic abdominal pain is a common symptom of digestive system diseases and gynecological diseases [26]. However, the prevalence of chronic abdominal pain in adolescents is seldom reported. This study showed a significant correlation between chronic abdominal pain and academic pressure. Mental stress has been shown to induce gastrointestinal ulcers and functional gastrointestinal disease [27]. In addition, academic pressure increases the probability that adolescents will develop unhealthy habits, such as tobacco and alcohol consumption [25], which greatly elevates the risk of developing digestive system diseases [28, 29]. Therefore, we believe that the effect of academic pressure on chronic abdominal pain is indirect, and the underlying mechanism may be related to long-term “stress” [30]. However, we also found that compared with chronic headache and musculoskeletal pain, the correlation between self-reported academic pressure and chronic abdominal pain is somewhat weaker. The reasons may include the following: (1) For a long time, people have been paying more attention to digestive system diseases [26]. The treatments for digestive system diseases are relatively effective and universally accessible [28]. The symptoms of chronic abdominal pain can be readily relieved using drugs and physical therapy. Therefore, the correlation between chronic abdominal pain and self-reported academic pressure cannot easily be detected. (2) The level of self-reported academic pressure that Chinese adolescents experience, while rather high, does not reach the severity of “stress”. Thus, the likelihood of causing chronic abdominal pain is fairly low [30, 31].

### NSP and LBP

Both NSP and LBP belong to musculoskeletal pain. Although the pathogenesis of NSP and LBP is different from the pathogenesis of chronic headache and abdominal pain [32], it is closely related to academic pressure. First, heavy schoolwork and fierce competition put much pressure on adolescents. Good mental health is proven to be able to reduce the prevalence of LBP [6]. The prevalence of NSP has been associated with depression, especially in female students [33]. Second, a heavy burden of learning often results in sleep deprivation in adolescents. Lack of sleep and poor sleep quality are independent risk factors for musculoskeletal disorders, especially in female students [34]. However, the results of this study are somewhat inconsistent with previous studies [35]. Third, a heavy learning burden is also reflected by prolonged study time, extended sedentary time, and lack of exercise. Long-term bent neck posture [36] and curvature of the spine [37] are closely related to the prevalence of NSP. Exercise stimulates blood circulation to the intervertebral discs [38] and enhances the endurance [39] and flexibility [23] of the lower back muscles, which increases the stability of the spine and exerts a protective effect against NSP and LBP.

In addition, a certain correlation has been established between headache and musculoskeletal pain (such as NSP, LBP, and elbow/knee/ankle joint pain) [22]. Headache is more closely related to NSP, while abdominal pain exhibits a stronger correlation with LBP [4]. The reason may be that chronic pain induces emotional and psychological changes that lead to enhanced sensitivity to NSP/LBP [22]. Abdominal pain is often accompanied by other types of chronic pain, such as muscle pain and headache [40]. Various types of musculoskeletal pain affect one another to a certain extent [31]. Therefore, studying the factors related to a specific type of chronic pain requires the elimination of those interaction effects. Besides, sleep problems and chronic pains may have a bilateral impact on each other, for example, the length, difficulty and quality of sleep may have an impact on different chronic pains, at the same time chronic pain (such as back pain) can influence sleeping by interfering process of getting asleep and the sleeping quality.

In summary, a detailed and objective lecture was given to the survey participants prior to the survey. Ample time was provided to complete the survey. Questionnaire items were subjected to repeated scrutiny based on the results of numerous investigations, discussions, and pre-surveys. The large sample size and high response rate to the questionnaire rendered the survey valuable for determining the correlation of risk factors. However, there are still some limitations as follows: (1) A self-assessment questionnaire was chosen for pain assessment. Because self-feeling rather than a scale was used to measure the pain levels, the reliability and validity were relatively low. To ensure standardization, the questionnaire was fully illustrated and explained prior to the launch of the formal survey. The pre-survey indicated that differences in the severity of pain were not significant among different individuals. Cases of severe pain were rather rare. (2) A cross-sectional study cannot establish causal relationships. (3) The scope of the survey was limited to the Shanghai area. Therefore, the survey may fail to represent the situation in other regions. However, Shanghai is an independent and inclusive area for the national college entrance exam. We believe that the conclusions of the survey will reflect the current state of Chinese adolescents to a certain extent. (4) Students suffering from chronic pain may be more willing to complete the questionnaire, while emotionally depressed students are more likely to avoid the survey. In addition, the mental state of the depressed students is rather susceptible to hints. (5) Considering varies caused by different methods and standards about pain and chronic pain, we can hardly achieve a convincible compare to previous studies. The prevalence rates are only considerable higher under the rules which set by us in this research. Therefore, the problem of *respondent bias* was present to a certain degree. During the interaction prior to the survey, a professional psychological consultant lectured the survey-participating adolescents and helped them to treat emotional problems correctly and objectively. Thus, the bias should be minimized.

In brief, the prevalence of chronic pain is rather high in Chinese adolescents. Musculoskeletal pain, represented by NSP and LBP, has the greatest impact on the Chinese adolescent health. The enormous academic pressure and the resulting changes in the living habits of adolescents are closely related to the prevalence of chronic pain.

## Conclusions

This study revealed the prevalence of chronic pain (headache, abdominal pain, NSP and LBP) in adolescents in China (Shanghai) and suggested that there is a relation between chronic pain and academic pressure, which needs to be explored in more depth in future studies. As the non-specific candidate risk factors, the heavy burden of learning which Chinese adolescents are bearing and self-reported academic pressure is closely related to the prevalence of chronic pain. Therefore, we call for measures to reduce the burden of learning on adolescents. In addition, we recommend further conducting cohort studies to explore the intrinsic correlation between various candidate risk factors such as self-reported academic pressure and chronic pain, and taking early preventive measures to reduce the prevalence of chronic pain in adolescents.

## Abbreviations

NSP: Neck and shoulder pain
LBP: Low back pain

## References

[1] Azevedo LF, Costa-Pereira A, Mendonca L, Dias CC, Castro-Lopes JM (2013). Chronic pain and health services utilization: Is there overuse of diagnostic tests and inequalities in nonpharmacologic treatment methods utilization?. Med Care.

[2] Ando S, Yamasaki S, Shimodera S, Sasaki T, Oshima N, Furukawa TA (2013). A greater number of somatic pain sites is associated with poor mental health in adolescents: A cross-sectional study. BMC Psychiatry.

[3] Latina R, Sansoni J, D’Angelo D, Di Biagio E, De Marinis MG, Tarsitani G (2013). Etiology and prevalence of chronic pain in adults: A narrative review. Prof Inferm.

[4] Fernandez-de-las-Penas C, Hernandez-Barrera V, Alonso-Blanco C, Palacios-Cena D, Carrasco-Garrido P, Jimenez-Sanchez S (2011). Prevalence of neck and Low back pain in community-dwelling adults in Spain: A population-based national study. Spine (Phila Pa 1976).

[5] Tan Z, Liang Y, Liu S, Cao W, Tu H, Guo L (2013). Health-related quality of life as measured with Eq-5d among populations with and without specific chronic conditions: A population-based survey in Shaanxi Province, China. PLoS One.

[6] Korovessis P, Repantis T, Baikousis A (2010). Factors affecting Low back pain in adolescents. J Spinal Disord Tech.

[7] Wulff C, Lindfors P, Sverke M (2011). Childhood general mental ability and midlife psychosocial work characteristics as related to mental distress, neck/shoulder pain and self-rated health in working women and men. J Occup Health.

[8] Diepenmaat AC, van der Wal MF, de Vet HC, Hirasing RA (2006). Neck/shoulder, low back, and Arm pain in relation to computer use, physical activity, stress, and depression among Dutch adolescents. Pediatrics.

[9] Hanvold TN, Veiersted KB, Waersted M (2010). A prospective study of neck, shoulder, and upper back pain among technical school students entering working life. J Adolesc Health.

[10] Hestbaek L, Leboeuf-Yde C, Kyvik KO (2006). Is comorbidity in adolescence a predictor for adult low back pain? A prospective study of a young population. BMC Musculoskelet Disord.

[11] Hestbaek L, Leboeuf-Yde C, Kyvik KO, Manniche C (2006). The course of Low back pain from adolescence to adulthood: Eight-year follow-up of 9600 twins. Spine (Phila Pa 1976).

[12] Erne C, Elfering A (2011). Low back pain at school: unique risk deriving from unsatisfactory grade in maths and school-type recommendation. Eur Spine J.

[13] Shan Z, Deng G, Li J, Li Y, Zhang Y, Zhao Q (2014). How schooling and lifestyle factors effect neck and shoulder pain? A cross-sectional survey of adolescents in china. Spine (Phila Pa 1976).

[14] Staes F, Stappaerts K, Lesaffre E, Vertommen H (2003). Low back pain in Flemish adolescents and the role of perceived social support and effect on the perception of back pain. Acta Paediatr.

[15] Roth-Isigkeit A, Thyen U, Stoven H, Schwarzenberger J, Schmucker P (2005). Pain among children and adolescents: restrictions in daily living and triggering factors. Pediatrics.

[16] Pollock CM, Harries RL, Smith AJ, Straker LM, Kendall GE, O’Sullivan PB (2011). Neck/shoulder pain is more strongly related to depressed mood in adolescent girls than in boys. Man Ther.

[17] Bensing JM, Hulsman RL, Schreurs KM (1999). Gender differences in fatigue: biopsychosocial factors relating to fatigue in men and women. Med Care.

[18] Chesterton LS, Barlas P, Foster NE, Baxter GD, Wright CC (2003). Gender differences in pressure pain threshold in healthy humans. Pain.

[19] Defrin R, Shramm L, Eli I (2009). Gender role expectations of pain is associated with pain tolerance limit but not with pain threshold. Pain.

[20] Widmalm SE, McKay DC, Radke JC, Zhang Y, Wang X, Wang M (2013). Gender differences in Low and high pain palpation thresholds in the Tmj and neck areas. Cranio.

[21] Blackwell L, Kaczynski K, Logan D (2009). Depression and school functioning in adolescents with chronic pain: The mediating role of pain-related catastrophizing. J Pain.

[22] Blaschek A, Milde-Busch A, Straube A, Schankin C, Langhagen T, Jahn K (2012). Self-reported muscle pain in adolescents with migraine and tension-type headache. Cephalalgia.

[23] Wober-Bingol C (2013). Epidemiology of migraine and headache in children and adolescents. Curr Pain Headache Rep.

[24] Milde-Busch A, Straube A, Heinen F, von Kries R (2012). Identified risk factors and adolescents’ beliefs about triggers for headaches: Results from a cross-sectional study. J Headache Pain.

[25] Lehmann S, Milde-Busch A, Straube A, von Kries R, Heinen F (2013). How specific are risk factors for headache in adolescents? Results from a cross-sectional study in Germany. Neuropediatrics.

[26] Coffelt TA, Bauer BD, Carroll AE (2013). Inpatient characteristics of the child admitted with chronic pain. Pediatrics.

[27] Gulewitsch MD, Enck P, Schwille-Kiuntke J, Weimer K, Schlarb AA (2013). Rome Iii criteria in parents’ hands: pain-related functional gastrointestinal disorders in community children and associations with somatic complaints and mental health. Eur J Gastroenterol Hepatol.

[28] Coates MD, Lahoti M, Binion DG, Szigethy EM, Regueiro MD, Bielefeldt K (2013). Abdominal pain in ulcerative colitis. Inflamm Bowel Dis.

[29] Ecevit CO, Ozgenc F, Yuksekkaya HA, Unal F, Arikan C, Yagci RV (2012). Peptic ulcer disease in children: an uncommon disorder with subtle symptomatology. Turk J Gastroenterol.

[30] Stanghellini V (2013). Perspectives on irritable bowel syndrome: Where have we been? Where are we now?. Expert Rev Gastroenterol Hepatol.

[31] Andersen LL, Clausen T, Carneiro IG, Holtermann A (2012). Spreading of chronic pain between body regions: prospective cohort study among health care workers. Eur J Pain.

[32] Huysmans MA, Ijmker S, Blatter BM, Knol DL, van Mechelen W, Bongers PM (2012). The relative contribution of work exposure, leisure time exposure, and individual characteristics in the onset of arm-wrist-hand and neck-shoulder symptoms among office workers. Int Arch Occup Environ Health.

[33] Watson KD, Papageorgiou AC, Jones GT, Taylor S, Symmons DPM, Silman AJ (2003). Low back pain in schoolchildren: The role of mechanical and psychosocial factors. Arch Dis Child.

[34] Auvinen JP, Tammelin TH, Taimela SP, Zitting PJ, Jarvelin MR, Taanila AM (2010). Is insufficient quantity and quality of sleep a risk factor for neck, shoulder and Low back pain? A longitudinal study among adolescents. Eur Spine J.

[35] Mongini F, Evangelista A, Milani C, Ferrero L, Ciccone G, Ugolini A (2012). An educational and physical program to reduce headache, neck/shoulder pain in a working community: A cluster-randomized controlled trial. PLoS One.

[36] Straker LM, O’Sullivan PB, Smith AJ, Perry MC (2009). Relationships between prolonged neck/shoulder pain and sitting spinal posture in male and female adolescents. Man Ther.

[37] Straker LM, O’Sullivan PB, Smith AJ, Perry MC, Coleman J (2008). Sitting spinal posture in adolescents differs between genders, but is Not clearly related to neck/shoulder pain: An observational study. Aust J Physiother.

[38] Korovessis P, Koureas G, Papazisis Z (2004). Correlation between backpack weight and way of carrying, sagittal and frontal spinal curvatures, athletic activity, and dorsal and Low back pain in schoolchildren and adolescents. J Spinal Disord Tech.

[39] Perich D, Burnett A, O’Sullivan P, Perkin C (2011). Low back pain in adolescent female rowers: A multi-dimensional intervention study. Knee Surg Sports Traumatol Arthrosc.

[40] Chelimsky G, Safder S, Chelimsky T (2012). Fgids in children are associated with many nonpsychiatric comorbidities: The tip of an iceberg?. J Pediatr Gastroenterol Nutr.

